# Dramatic Increase in Glycerol Biosynthesis upon Oxidative Stress in the Anaerobic Protozoan Parasite *Entamoeba histolytica*

**DOI:** 10.1371/journal.pntd.0001831

**Published:** 2012-09-27

**Authors:** Afzal Husain, Dan Sato, Ghulam Jeelani, Tomoyoshi Soga, Tomoyoshi Nozaki

**Affiliations:** 1 Department of Parasitology, National Institute of Infectious Diseases, Tokyo, Japan; 2 Department of Parasitology, Gunma University Graduate School of Medicine, Maebashi, Japan; 3 Institute for Advanced Biosciences, Keio University, Tsuruoka, Japan; 4 Department of Biochemistry and Integrative Medical Biology, School of Medicine, Keio University, Tokyo, Japan; 5 Graduate School of Life and Environmental Sciences, University of Tsukuba, Tsukuba, Japan; University of Tokyo, Japan

## Abstract

*Entamoeba histolytica*, a microaerophilic enteric protozoan parasite, causes amebic colitis and extra intestinal abscesses in millions of inhabitants of endemic areas. Trophozoites of *E. histolytica* are exposed to a variety of reactive oxygen and nitrogen species during infection. Since *E. histolytica* lacks key components of canonical eukaryotic anti-oxidative defense systems, such as catalase and glutathione system, alternative not-yet-identified anti-oxidative defense strategies have been postulated to be operating in *E. histolytica*. In the present study, we investigated global metabolic responses in *E. histolytica* in response to H_2_O_2_- and paraquat-mediated oxidative stress by measuring charged metabolites on capillary electrophoresis and time-of-flight mass spectrometry. We found that oxidative stress caused drastic modulation of metabolites involved in glycolysis, chitin biosynthesis, and nucleotide and amino acid metabolism. Oxidative stress resulted in the inhibition of glycolysis as a result of inactivation of several key enzymes, leading to the redirection of metabolic flux towards glycerol production, chitin biosynthesis, and the non-oxidative branch of the pentose phosphate pathway. As a result of the repression of glycolysis as evidenced by the accumulation of glycolytic intermediates upstream of pyruvate, and reduced ethanol production, the levels of nucleoside triphosphates were decreased. We also showed for the first time the presence of functional glycerol biosynthetic pathway in *E. histolytica* as demonstrated by the increased production of glycerol 3-phosphate and glycerol upon oxidative stress. We proposed the significance of the glycerol biosynthetic pathway as a metabolic anti-oxidative defense system in *E. histolytica*.

## Author Summary

During the course of infection, trophozoites of *E. histolytica* need to cope with the oxidative stress in order to survive under the oxidative environment of its host. As a result of the absence of the key eukaryotic anti-oxidative defense system, it needs to employ novel defense strategies. Several studies such as transcriptomic profiling of trophozoites exposed to oxidative stress, and biochemical and functional analysis of individual proteins has been done in the past. Since, oxidative stress damages several metabolic enzymes, and modulate expression of many genes, it is important to analyze the detailed metabolomic response of *E. histolytica* upon oxidative stress to understand the role of metabolism in combating oxidative stress. In the present study, we demonstrated that oxidative stress causes glycolytic inhibition and redirection of metabolic flux towards glycerol production, chitin biosynthesis, and the non-oxidative branch of the pentose phosphate pathway.

## Introduction

Organisms are exposed to a variety of reactive oxygen and nitrogen species (ROS and RNS) that cause damage to key biomolecules such as proteins, lipids and DNA, and lead to cellular dysfunction [1]–[3]. In many cases, oxidative stress also induces genetic programs such as apoptosis, which ultimately leads to cell death [3]–[6]. In order to protect themselves against these oxidative damages, cells utilize effective defense mechanisms including antioxidant enzymes and free radical scavengers [7]. It is now well established that most microbial and higher eukaryotic cells have the ability to cope with oxidative stress by altering global expression of antioxidants and other metabolic enzyme encoding genes at transcriptional and post-transcriptional levels. However, it is becoming increasingly acceptable that post-translational mechanisms are also important players of cellular responses to oxidative stress. For instance, it has recently been shown that upon oxidative stress, metabolic flux is redirected from glycolysis to the pentose phosphate pathway to synthesize reductant for antioxidant metabolism [8].

*Entamoeba histolytica* is a protozoan parasite that causes dysentery and extra intestinal abscesses in millions of inhabitants of endemic areas [9]. *E. histolytica* trophozoites are microaerophilic, and have been shown to consume oxygen, and tolerate low levels of oxygen pressure [10]. In addition, they are also exposed to various reactive oxygen or nitrogen species (ROS and RNS) during tissue invasion, colonization, and extra intestinal propagation [9], [11]. *E. histolytica* lacks most of the components, such as catalase, reduced glutathione, and the glutathione-recycling enzymes [12]–[14], involved in the usual antioxidant defense mechanisms in aerobic organisms. *E. histolytica* also lacks glucose 6-phosphate dehydrogenase (G6PD), and thus functional pentose phosphate pathway (PPP) is absent [12]. However, the genome of *E. histolytica* encodes several proteins such as peroxiredoxin, superoxide dismutase, rubrerythrin, hybrid-cluster protein, and flavo-di-iron proteins for detoxification of ROS and RNS [12], [15], [16]. In addition, *E. histolytica* also possesses a cryptic pyridine nucleotide trans-hydrogenase, which utilizes the electrochemical proton gradient across the membrane to drive NADPH synthesis from NADH [17], [18].

The precise nature of the molecular response to oxidative stress in *E. histolytica* is poorly understood. Several genes have shown to be differentially expressed in *E. histolytica* subjected to oxidative or nitrosative stress [19]. However, the specific regulatory elements of this transcriptomic response are yet to be uncovered. It has been shown that in bacteria and yeast, the expression of genes involved in antioxidative defense mechanisms is partially induced upon oxidative stress [20], [21]. However, like most eukaryotic cells, the transcription of genes encoding enzymes capable of neutralizing ROS and RNS is generally not increased in *E. histolytica* subjected to oxidative stress [19]. In yeast and bacteria, post transcriptional and post translational studies have recently demonstrated that a large number of metabolic enzymes are targeted by oxidative stress that led to redirection of metabolic flux [8]. What little is known in *E. histolytica* relates to oxidative stress-dependent inactivation of some enzymes involved in central energy metabolism [22]. However, it remains unknown how *E. histolytica* and other organisms that lack fully functional PPP such as *Trichomonas* and *Giardia* [23], [24] respond to oxidative stress at the metabolic level.

In order to get insight into the dynamics of metabolic changes employed either to bypass damaged enzymes or to support adaptive responses to cope up with oxidative stress, we undertook detailed metabolomic study of *E. histolytica* upon oxidative stress. We employed capillary electrophoresis time-of-flight mass spectrometry (CE-TOFMS) [25]–[27] to simultaneously detect and quantify hundreds of intermediary metabolites of primary metabolism. The major advantages of CE-TOFMS analysis include its extremely high resolution and ability to simultaneously quantify a variety of charged low–molecular weight compounds [25]–[27]. In the present study, we highlight several unanticipated oxidative stress-mediated metabolomic changes, and discuss their relevance in relation to oxidative stress in *E. histolytica*. In addition to metabolomic analysis, the activities of several enzymes that are of potential importance in regulating observed metabolomic changes are evaluated. The obtained data are discussed in terms of the correlations between the metabolome and posttranslational inactivation of key metabolic enzymes.

## Materials and Methods

### Chemicals and reagents

All chemicals of analytical grade were purchased from either Wako (Tokyo, Japan) or Sigma-Aldrich (Tokyo, Japan) unless otherwise mentioned. 2′, 7′-Dichlorodihydrofluorescein di-acetate (2′, 7′-DCF-DA) was purchased from Invitrogen. Stock solutions of metabolite standards (1–100 mmol/L) for CE-MS analysis were prepared in either Milli-Q water, 0.1 mol/L HCl, or 0.1 mol/L NaOH. A mixed solution of the standards was prepared by diluting stock solutions with Milli-Q water immediately before CE-TOFMS analysis.

### Microorganisms and cultivation

Trophozoites of the *E. histolytica* clonal strain HM-1: IMSS cl 6 were maintained axenically in Diamond's BI-S-33 medium at 35.5°C, as described previously [28], [29]. Trophozoites were harvested in the late-logarithmic growth phase 2–3 days after the inoculation of medium with one-thirtieth to one-twelfth of the total culture volume.

### Induction of oxidative stress and metabolite extraction

*E. histolytica* trophozoites were cultivated in standard BI-S-33 medium until late logarithmic phase in 36 ml culture flaks, and then culture medium was replaced with fresh and warm culture medium. To induce oxidative stress, paraquat (PQ) or H_2_O_2_ were added to the final concentration of 1 or 0.4 mM, respectively, and culture was continued for next 1, 3, 6, or 12 h. Intracellular metabolites were extracted as previously described with some modifications [30], [31]. Approximately 1.5×10^6^ trophozoites from each condition were harvested and immediately suspended in 1.6 ml of −75°C methanol to quench metabolic activity. To ensure that experimental artifacts such as ion suppression did not lead to misinterpretation of metabolite levels, internal standards, 2-(*N*-morpholino) ethanesulfonic acid, methionine sulfone, and D-camphor-10-sulfonic acid were added to every sample. The samples were then sonicated for 30 second and mixed with 1.6 ml of chloroform, and 640 µl of deionized water. After vortexing, the mixture was centrifuged at 4,600× *g* at 4°C for 5 min. The aqueous layer (1.6 ml) was filtrated using an Amicon Ultrafree-MC ultrafilter (Millipore Co., Massachusetts, USA) and centrifuged at 9,100× *g* at 4°C for approximately 2 h. The filtrate was dried and preserved at −80°C until mass spectrometric analysis [30], [32]. Prior to the analysis, metabolic extracts was dissolved in 20 µl of de-ionized water containing reference compounds (200 µmol/L each of 3-aminopyrrolidine and trimesic acid).

### Instrumentation and capillary electrophoresis-time-of-flight mass spectrometry (CE-TOFMS) conditions

CE-TOFMS was performed using an Agilent CE Capillary Electrophoresis System equipped with an Agilent 6210 Time-of-Flight mass spectrometer, Agilent 1100 isocratic HPLC pump, Agilent G1603A CE-MS adapter kit, and Agilent G1607A CE-ESI-MS sprayer kit (Agilent Technologies, Waldbronn, Germany). The system was controlled by Agilent G2201AA ChemStation software for CE. Data acquisition was performed by Analyst QS software for Agilent TOF (Applied Biosystems, CA, USA; MDS Sciex, Ontario, Canada).

### CE-TOFMS conditions for cationic metabolite analysis

Cationic metabolites were separated in a fused-silica capillary (50 µm i.d.×100 cm total length) filled with 1 mol/L formic acid as the reference electrolyte [33]. Sample solution (∼3 nL) was injected at 50 mbar for 3 s, and a positive voltage of 30 kV was applied. The capillary and sample trays were maintained at 20°C and below 5°C, respectively. Sheath liquid composed of methanol/water (50% v/v) that contained 0.1 µmol/L hexakis (2,2-difluoroethoxy) phosphazene was delivered at 10 µL/min. ESI-TOFMS was operated in the positive ion mode. The capillary voltage was set at 4 kV and a flow rate of nitrogen gas (heater temperature 300°C) was set at 10 psig. For TOFMS, the fragmenter voltage, skimmer voltage, and octapole radio frequency voltage (Oct RFV) were set at 75, 50, and 125 V, respectively. An automatic recalibration function was performed using two reference masses of reference standards; protonated ^13^C methanol dimer (*m/z* 66.063061) and protonated hexakis (2,2-difluoroethoxy) phosphazene (*m/z* 622.028963), which provided the lock mass for exact mass measurements. Exact mass data were acquired at the rate of 1.5 cycles/s over a 50 to 1,000 *m/z* range.

### CE-TOFMS conditions for anionic metabolite analysis

Anionic metabolites were separated in a cationic-polymer–coated COSMO(+) capillary (50 µm i.d.×110 cm) (Nacalai Tesque) filled with 50 mmol/L ammonium acetate solution (pH 8.5) as the reference electrolyte [34], [35]. Sample solution (∼30 nL) was injected at 50 mbar for 30 s and a negative voltage of −30 kV was applied. Ammonium acetate (5 mmol/L) in 50% methanol/water (50% v/v) that contained 0.1 µmol/L hexakis (2,2-difluoroethoxy) phosphazene was delivered as sheath liquid at 10 µL/min. ESI-TOFMS was operated in the negative ion mode. The capillary voltage was set at 3.5 kV. For TOFMS, the fragmenter voltage, skimmer voltage, and Oct RFV were set at 100, 50, and 200 V, respectively [35]. An automatic recalibration function was performed using two reference masses of reference standards: deprotonated ^13^C acetate dimer (*m/z* 120.038339) and acetate adduct of hexakis (2,2-difluoroethoxy) phosphazene (*m/z* 680.035541). The other conditions were identical to those used for the cationic metabolome analysis.

### CE-TOFMS data processing

Raw data were processed using the in-house software Masterhands [36]. The overall data processing flow consisted of the following steps: noise-filtering, baseline-removal, migration time correction, peak detection, and integration of peak area from a 0.02 *m/z*-wide slice of the electropherograms. This process resembled the strategies employed in widely used data processing software for LC-MS and GC-MS data analysis, such as MassHunter (Agilent Technologies) and XCMS [37]. Subsequently, accurate *m/z* values for each peak were calculated by Gaussian curve fitting in the m/z domain, and migration times were normalized using alignment algorithms based on dynamic programming [25], [38]. All target metabolites were identified by matching their *m/z* values and normalized migration times with those of standard compounds in the in-house library.

### Assay of enzymatic activities

In order to assay activities of various enzymes of central energy metabolism, *E. histolytica* trophozoites were exposed to PQ as described above. Trophozoites were harvested by chilling on ice, centrifuging at 450 g, and washing twice with NaCl/Pi at pH 7.4. Trophozoites were then lysed in NaCl/Pi (pH 7.0) by freezing–thawing. The insoluble materials were eliminated by centrifugation at 15 000× *g* for 10 min. The activities of several glycolytic enzymes in the soluble fraction were determined as described previously [22].

### Quantitation of Reactive Oxygen Species

Fluorescence spectrophotometry was used to measure the production of intracellular reactive oxygen species using 2′, 7′-DCF-DA as a probe as previously described [30]. Briefly, untreated or 1 mM PQ treated *E. histolytica* trophozoites were washed in PBS, and 5.0×10^5^ cells were then incubated in 1 ml of PBS containing 20 µM of 2′, 7′-DCF-DA for 30 min at 35.5°C in the dark. The intensity of fluorescence was immediately read at excitation and emission wavelengths of 492 and 519 nm, respectively.

### Glycerol, ethanol and acetate quantitation

To estimate the production of glycerol, ethanol or acetate, approximately 10^7^ normally cultured or stressed trophozoites (1 mM PQ for 12 h) were suspended in 1.0 mL of NaCl/Pi (117 mM NaCl, 2.3 mM KCl, 8.5 mM Na_2_HPO_4_, 1.7 mM KH2PO4; pH 7.4) containing 1% glucose (w/v), and incubated in a water bath at 35.5°C. After 30 min, the cells were quickly harvested by centrifugation at 500× *g* for 5 min. Supernatants were used for the estimation of ethanol, acetate and glycerol. The cellular pellets were extracted with perchloric acid, as described earlier and used for the estimation of intracellular glycerol. Ethanol and glycerol were determined using ethanol or glycerol estimation kits from Biovision (Mountain View, CA). Acetate was estimated by using acetate kinase through the coupling reaction of this enzyme with pyruvate kinase, and lactate dehydrogenase [30]. Acetate kinase generates ADP and acetyl-phosphate from acetate and ATP. The ADP production was coupled with the oxidation of NADH (ε_340_ = 6.22 mM^−1^ cm^−1^) through pyruvate kinase and lactate dehydrogenase. The standard reaction mixture contained 50 mM of Tris-Cl, 3 units each of acetate kinase, pyruvate kinase and lactate dehydrogenase, 0.5 mM ATP, 0.3 mM NADH, and 0.4 mM phosphoenolpyruvate (PEP). Reactions were initiated by the addition of supernatant containing acetate, and optical absorbance was read at 340 nm on a Shimadzu spectrophotometer.

## Results and Discussion

### Oxidative stress causes drastic changes in the metabolome of *E. histolytica*

**Figure 1:**
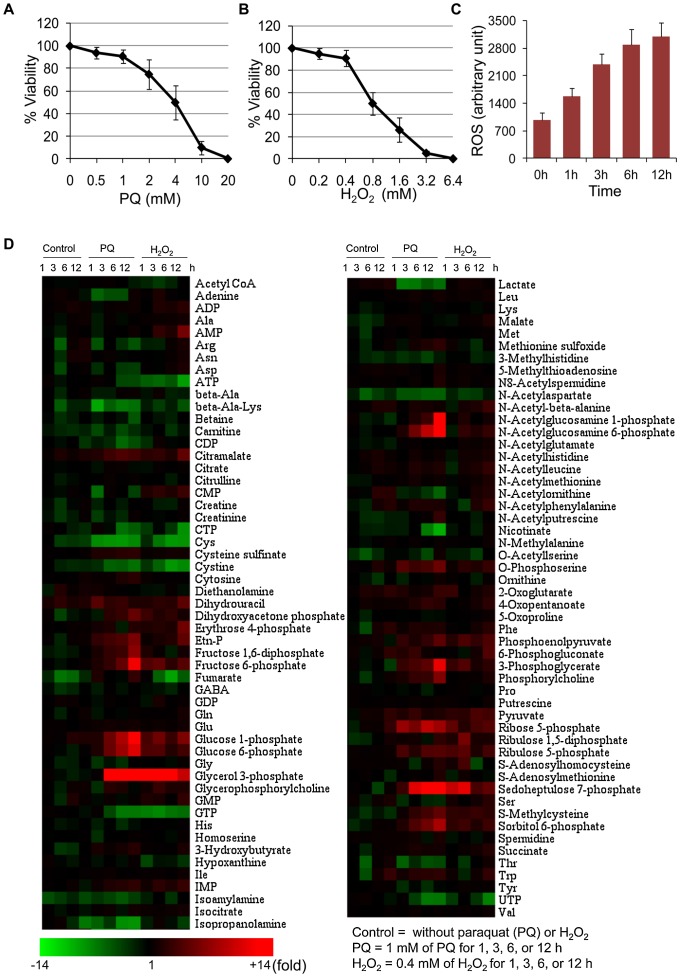
PQ- or H_2_O_2_-mediated oxidative stress causes global metabolic changes. (**A–B**) Survival of *E. histolytica* trophozoites after 12 h of treatment with varying concentrations of PQ (**A**) or H_2_O_2_ (**B**). The average viability (%) ± standard deviation (SD, error bars) at various concentrations of PQ or H_2_O_2_ is shown. (**C**) Effect of 1 mM of PQ treatment for 1, 3, 6, or 12 h on the intracellular level of reactive oxygen species. Average level of 2′, 7′-DCF-DA fluorescence (arbitrary units) ±S.D. (error bars) in 5×10^5^ cells is shown (**D**) Heat map produced by hierarchical clustering of metabolite profiles obtained from CE-TOFMS analysis. Rows correspond to metabolites and columns correspond to the duration of treatment. Metabolite levels are expressed as fold change with respect to time 0 h. Shades in red and green indicate increase or decrease in the levels of metabolites, respectively, according to the scale bar shown at the bottom.

**Figure 2:**
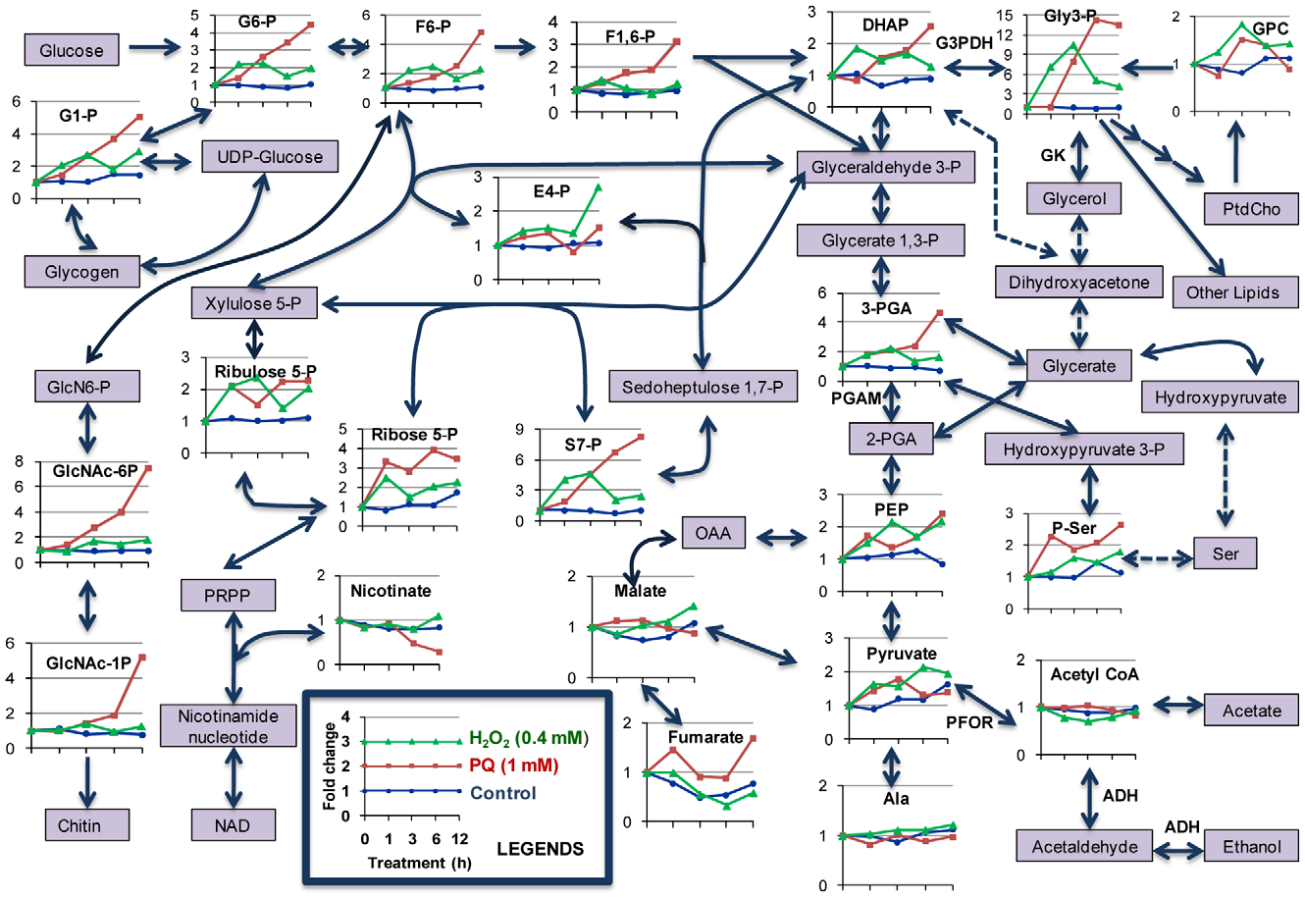
Oxidative stress causes drastic changes in central energy metabolism. The average fold change ± SD (error bars) of metabolites in untreated (control), PQ (1 mM), or H_2_O_2_ (0.4 mM) treated trophozoites with respect to time 0 h is shown. The enzymes discussed in the text are also shown in bold letters. Abbreviations are: G6-P, glucose 6-phosphate; G1-P, glucose 1-phosphate; F6-P, fructose 6-phosphate; F1,6-P, fructose 1,6-diphosphate; DHAP, dihydroxyacetone phosphate; GPC, glycerophosphocholine; Gly 3-P, glycerol 3-phosphate; 3-PGA, 3-phosphoglycerate; 2-PGA, 2-phosphoglycerate PEP, phosphoenolpyruvate; and P-Ser; O-phosphoserine; E4-P, erythrose 4-phosphate; S7-P, Sedoheptulose 7-phosphate; OAA; oxaloacete; PtdCho, phosphatidylcholine; PFOR, pyruvate:ferredoxin oxidoreductase; PGAM, phosphoglycerate mutase; G3PDH, glycerol 3-phosphate dehydrogenase; GK, glycerol kinase.

In order to study metabolomic responses of *E. histolytica* upon oxidative stress, we exposed amebic trophozoites to hydrogen peroxide (H_2_O_2_) or paraquat (PQ), which are widely used to study oxidative stress in various organisms. Peroxide stress was generated by directly adding H_2_O_2_ to a desired concentration, whereas superoxide stress was generated indirectly during metabolism of exogenously added PQ [Fig. 1C]. In order to identify conditions to monitor short- to long-term (∼12 h) responses, in which trophozoites were stressed, and still viable, we estimated the survival of trophozoites upon treatment with varying concentrations of PQ (0.25–8.0 mM) or H_2_O_2_ (0.2–6.4 mM) for 12 h. Trophozoites exposed to 1 mM of PQ or 400 µM of H_2_O_2_ for 12 h showed a viability of ≥90%, while trophozoites exposed to higher concentrations showed lesser viability (Fig. 1, A and B). Thus, we chose 1 mM of PQ or 400 µM of H_2_O_2_, and exposed trophozoites for 1, 3, 6, or 12 h to these oxidants, and compared the metabolome with that of untreated trophozoites. Approximately 100 metabolites were quantitated by using CE-TOFMS and enzymatic procedures as described in Materials and Methods. Both PQ- and H_2_O_2_-mediated oxidative stress resulted in a drastic modulation of several metabolites involved in glycolysis and its associated pathways, and amino acid, nucleotide, and phospholipid metabolism. The changes in the relative levels of quantified metabolites upon oxidative stress as visualized by hierarchical clustering are shown in Fig. 1D. Time course data for metabolites or pathways of particular interest are shown in Fig. 2–4. Metabolomic response to both PQ and H_2_O_2_ showed similarity in terms of metabolites changed; however, time kinetics of changes in the modulated metabolites is clearly different (Fig. 1D). In addition, PQ- and H_2_O_2_-specific responses were also detected. In general, when compared to PQ treatment, H_2_O_2_ treatment caused rapid but mild changes in the metabolome. These differences in the kinetics and magnitude of metabolomic data are likely attributable to nature of these oxidants. H_2_O_2_ is an oxidizing agent itself, and thus results in faster appearance of the changes in the metabolome, whereas PQ indirectly generates reactive oxygen species resulting in delayed metabolomic response.

**Figure 3 pntd-0001831-g003:**
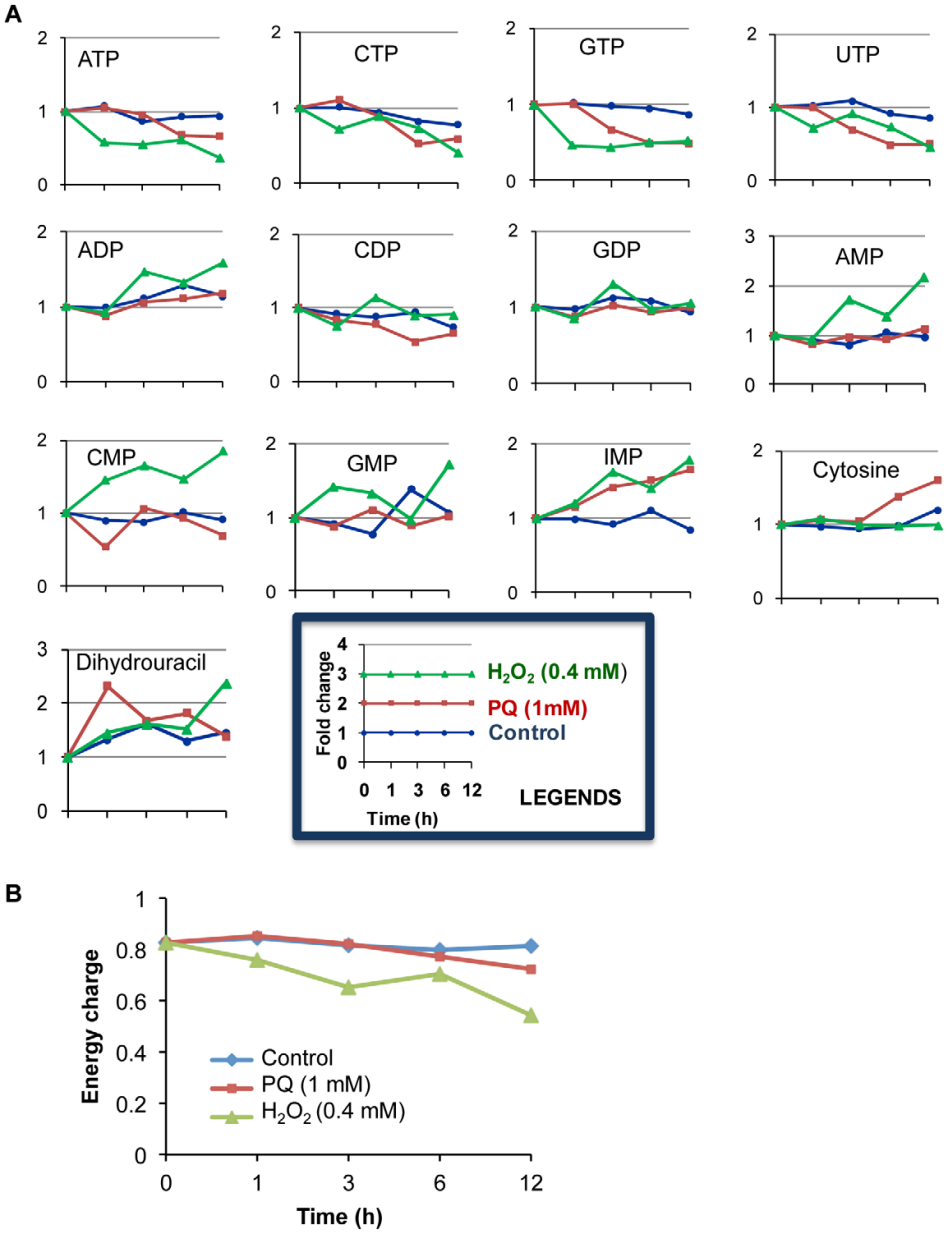
Oxidative stress causes energy depletion. (**A**) The average fold change ± SD (error bars) of the nucleotides at various time points during PQ- or H_2_O_2_-mediated oxidative stress. (**B**) Adenylate energy charge of the cell, which is calculated by the equation, [(ATP)+1/2(ADP)]/[(ATP)+(ADP)+(AMP)] during the course of oxidative stress.

**Figure 4:**
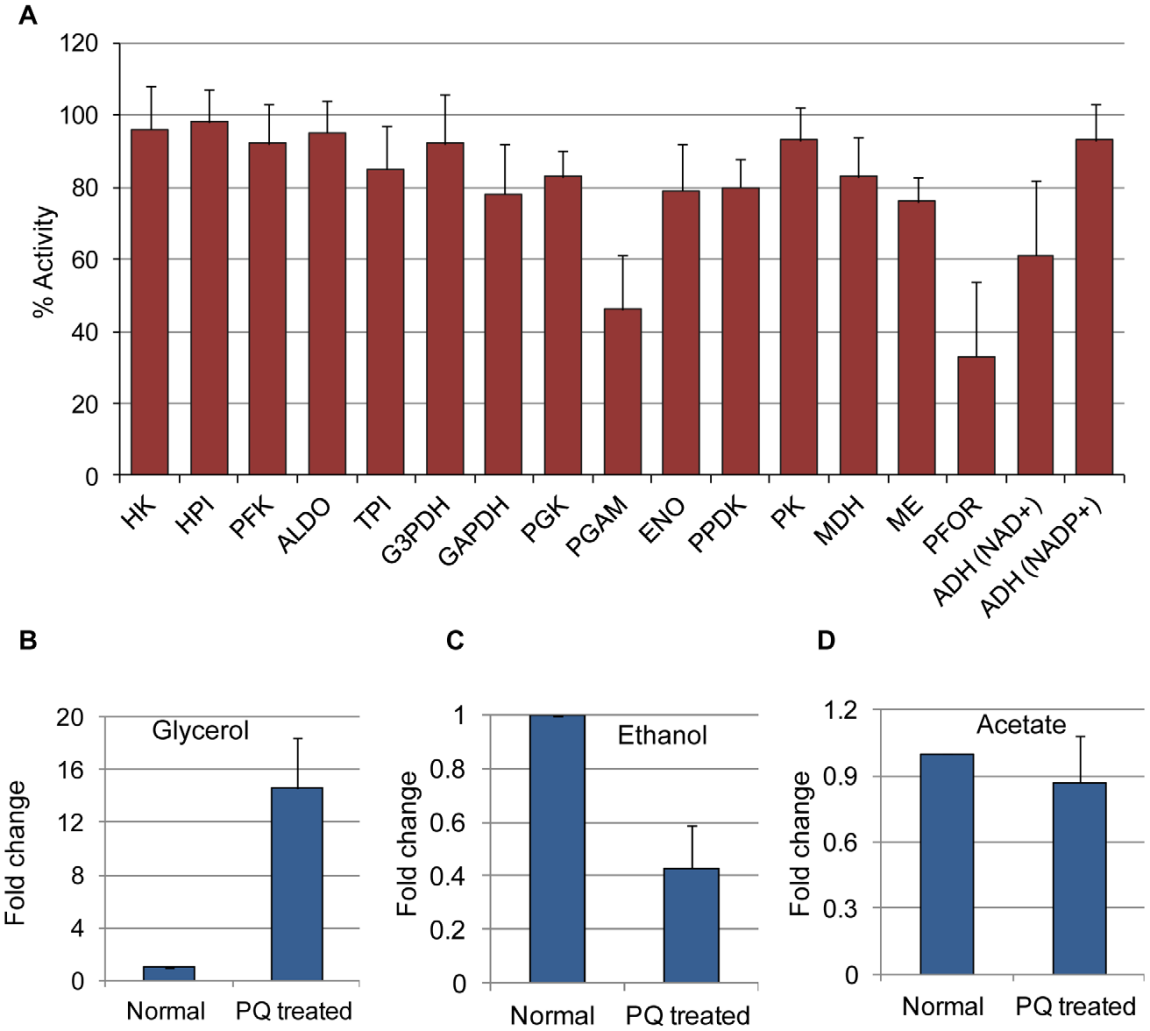
Oxidative stress inactivates glycolytic enzymes and redirects glycolytic flux towards glycerol synthesis. (**A**) Activities of enzymes involved in the central energy metabolism upon PQ (1 mM for 12 h) treatment. Enzymatic activities are expressed as percentage relative to untreated trophozoites. (**B–D**) The average fold change ± SD (error bars) of glycerol, ethanol, and acetate in untreated (control) or PQ-treated (1 mM for 12 h) trophozoites are shown. Abbreviations are: HK, Hexokinase; HPI, Hexose phosphate isomerase; PFK, Phosphofructokinase; ALDO, Aldolase; TPI, Triose-phosphate isomerase; GAPDH, glyceraldehyde 3-P dehydrogenase; PGK, Phosphoglycerate kinase; ENO, Enolase; PPDK, Pyruvate phosphate dikinase; PK, Pyruvate kinase; MDH, Malate dehydrogenase; ME, malic enzyme; ADH, Alcohol dehydrogenase.

### Central energy metabolism is repressed by oxidative stress

Like *G. lamblia* and *T. vaginalis*, *E. histolytica* is also microaerophilic, and lacks features of aerobic eukaryotic metabolism, including TCA cycle and oxidative phosphorylation, and generates energy exclusively by substrate level phosphorylation and fermentation [12], [39]. Among the metabolites that were measured by CE-TOFMS-based metabolomic analysis, oxidative stress caused modulation of several metabolites involved in central energy metabolism. We observed a general increase in the abundances of the metabolites involved in of the glycolysis and its associated pathways (Fig. 1D and 2). We observed a 2–6-fold increase in most of the glycolytic intermediates from glucose 6-phosphate (G 6-P) to pyruvate, i.e. fructose 6-phosphate (F 6-P), fructose 1, 6-diphosphate (F 1,6-P), dihydroxyacetone phosphate (DHAP), 3-phosphoglycric acid (3-PGA), and phosphoenolpyruvate, upon oxidative stress caused by both PQ and H_2_O_2_. We also expected a similar increment in the level of glyceraldehyde 3-phosphate. However, due to its high turnover or low stability, the metabolite was undetected using CE-TOFMS. The accumulation of these glycolytic intermediates suggests that there is a relative decrease in the flux through pathways downstream of glycolysis. In contrast to the significant increase in the abundance of glycolytic intermediates upstream of pyruvate upon oxidative stress, we observed an approximately 2-fold decrease in ethanol, the major end product of glucose catabolism in *E. histolytica* (Fig. 4C, see below), supporting the premise that the glycolytic flux downstream of pyruvate was indeed repressed. However, no significant change in acetate production was observed (Fig. 4D, see below). In addition, we also found that upon oxidative stress the accumulated glycolytic metabolites were re-routed towards pathways associated with glycolysis. For example, the metabolites are re-directed towards chitin biosynthetic pathway, glycerol production via glycerol 3-phosphate, and O-phosphoserine, an immediate precursor of L-serine. Accumulation of glycolytic intermediates hints repression of glycolysis as result of free radical-mediated inactivation of key glycolytic enzymes. Accumulation of glycolytic intermediates such as pyruvate, glucose 6-phosphate and fructose 6-phosphate, and repression of glycolytic flux upon oxidative or nitrosative stress has previously been shown in *E. histolytica* [40], [41]. Ramos-Martínez, E. *et al.* [40] reported accumulation of G 6-P, F 6-P, and DHAP, and decrement in ATP and ethanol production upon SNP (sodium nitroprusside, nitric oxide producer) mediated apoptosis in *E. histolytica*. Similarly, an acumulation of G 6-P, F 6-P, and pyruvate, and decrement in ATP and ethanol was also reported in *E. histolytica* exposed to hyperbaric oxygen [41]. Although the nature of changes in selected glycolytic intermediates and ATP upon oxidative or nitrosative stress reported in the previous studies was similar to that in the present study, the magnitude of these changes varies. For example, Ramos-Martínez E. *et al.* [41] reported a 6–7 fold increment in the level of pyruvate, whereas niether PQ nor H_2_O_2_ treatment caused >2 fold change in our study. These differences are likely due to chemical nature, concentrations and the exposure time of oxidants, experimental design, and the methods of metabolite quantitation. We should also emphasize that our CE-TOFMS-based method to quantitate charged metabolites is far more sensitive than the enzyme-based quantitation methods used previously.

### Non-oxidative pentose phosphate pathway is replenished by oxidative stress

The pentose phosphate pathway (PPP) is, in general, an anabolic pathway that generates ribose-5-phosphate for the synthesis of the nucleotides and nucleic acids, and reducing equivalents, in the form of NADPH, for reductive biosynthetic processes. PPP is widely distributed, and found in most prokaryotes and eukaryotes. However, PPP is not functional in *E. histolytica*, which lacks glucose 6-phosphate dehydrogenase (G6PDH) and transaldolases [12]. As shown in Fig. 1D and 2, most intermediates of the non-oxidative branch of PPP were increased upon oxidative stress. Erythrose 4-phosphate (E4-P), ribulose 5-phosphate (ribulose 5-P), and ribose 5-phosphate (ribose 5-P) showed 1.5 to 4-fold increments. Moreover, sedoheptulose 7-phosphate (S7-P) showed a maximum increment of 5 to 9 fold upon PQ or H_2_O_2_ treatment. Changes in all of these metabolites appear earlier upon H_2_O_2_ treatment, compared to PQ-mediated oxidative stress.

As described above, most of the intermediates of glycolysis upstream of acetylCoA were accumulated upon oxidative stress. These data indicate that oxidative stress blocks glycolysis, while glucose uptake continues, leading to accumulation and redirection of the metabolic flux from glycolysis to associated pathways. *E. histolytica* lacks G6PDH and transaldolase; however, it possesses an alternative hexose-pentose interconversion pathway that does not require transaldolases, but depends on three enzymes: phosphofructokinase, transketolase, and aldolase [42]. Thus, our data validate the previous reports of presence and functionality of such non-oxidative hexose-pentose inter-conversion pathway in *E. histolytica* [42].

It has been shown in yeast that oxidative stress redirects metabolic flux from glycolysis to PPP, leading to generation of NADPH [8]. However, such a redirection of metabolic flux in *E. histolytica* does not generate NADPH as it lacks the oxidative branch of PPP; however, this redirection results in the accumulation of several intermediates of non-oxidative branch of PPP using the alternative hexose-pentose interconversion pathway [42]. These data indicate an additional NADPH-independent role of the non-oxidative branch of PPP. A recent report has indeed shown such an NADPH-independent role of non-oxidative PPP intermediates in transcriptional re-programming after oxidative stress [43]. It was shown that the blockade of or the increment in the production of sedoheptulose 7-phosphate and other intermediates of the non-oxidative branch of PPP decreased or increased tolerance against oxidants, respectively [43]. Although a protective role of these intermediates was previously discussed in yeast [43], it needs to be further tested whether it is also a case for *E. histolytica* because it lacks oxidative branch of the pentose phosphate pathway. In addition, ribose 5-phosphate synthesized in this pathway is also a precursor of NAD, which is used by NAD kinase to synthesize NADP ([44], and Jeelani et al. unpublished). NADP is further used by pyridine nucleotide trans-hydrogenase to synthesize NADPH using electrochemical proton gradient [17].

### Activation of chitin biosynthetic pathway

*E. histolytica* exists in two morphologically distinct forms during its life cycle: the proliferative, non-infective, but pathogenic trophozoite form, and the dormant non-pathogenic, but infective, cyst form. The surface of the cysts is protected with the cell wall composed of chitin [45], [46]. We observed activation of the chitin biosynthetic pathway upon oxidative stress. Two key intermediates of this pathway, namely N-acetylglucosamine 6-phosphate (GlcNAc 6-P) and N-acetylglucosamine 1-phosphate (GlcNAc 1-P) were increased upon oxidative stress. However, PQ-mediated oxidative stress caused more dramatic increments (5–8-fold), compared to H_2_O_2_ (1.5–2.0-fold). These results suggest that oxidative stress may serve as an environmental stimulus to trigger encystation. Indeed, H_2_O_2_ at the concentration 10-fold higher than that used in this study (4 vs. 0.4 mM) was shown to induce glucosamine 6-phosphate isomerase, an enzyme that interconverts GlcNAc 6-P and GlcNAc 1-P, and formation of cyst-like structures in *E. histolytica* [47]. In addition, oxidative stress has also been shown to induce differentiation of *G. lamblia* [48]. One of the possible reasons of the induction of the chitin biosynthetic pathway may be that trophozoite needs to transform into the dormant cyst form, which is tolerant to the adverse external environments. In addition, it was also suggested that chitin oligosaccharides can also serve as potent antioxidants by scavenging free radicals [49].

### Oxidative stress modulates nucleotide metabolism

As glycolysis is the major source of energy generation in *E. histolytica*, a reduced glycolytic flux is expected to cause depletion of the energy content in the trophozoites. As expected, the levels of the nucleoside triphosphates, ATP, GTP, UTP, and CTP, were significantly decreased upon oxidative stress (Fig. 3A). In contrast, the levels of nucleoside monophosphates (AMP, CMP, GMP, and IMP) were increased upon H_2_O_2_ stress, in a manner opposite to the decrement in their corresponding triphosphates counterparts. The levels of nucleoside monophosphates upon PQ-mediated oxidative stress remain unchanged except IMP, which was increased in a time-dependent manner. We also examined the average adenylate energy charge during oxidative stress (Fig. 3B). Energy charge was decreased upon oxidative stress; however, the decrement was more prominent by H_2_O_2_ stress compared to PQ-mediated oxidative stress. The decrement in nucleoside triphosphates and energy charge is consistent with the repression of glycolysis, which is also evident by the accumulation of glycolytic intermediates, and decreased ethanol production (Fig. 2 and 4C). We also quantitated the levels of nicotinamide nucleotides in trophozoites exposed to PQ-mediated oxidative stress (data not shown). The level of NAD was decreased, whereas the level of NADP was increased upon paraquat treatment for 12 h. However, the levels of reduced form of these nicotinamide nucleotides only slightly decreased (data not shown).

### Modulation of sulfur-containing amino acid metabolism

Amino acid metabolism in *E. histolytica* is important in generating ATP by amino acid catabolism [50]. In addition, *E. histolytica* possesses unique pathways for the synthesis and degradation of sulfur-containing amino acids and their derivatives. For example, it lacks functional trans-sulfuration pathways, but possesses methionine γ-lyase for the degradation of sulfur-containing amino acids [51]–[54]. Furthermore, it also possesses unique *de novo* methanethiol or sulfur (sulfide) assimilatory pathways for the synthesis of S-methylcysteine or L-cysteine, respectively [30], [51], [52]. As amino acid metabolism plays an important role in the biology of *E. histolytica*, we also examined the effects of oxidative stress on the amino acid level. Both PQ and H_2_O_2_-mediated oxidative stress led to decrements in L-cysteine and L-cystine, and slight increment in cysteine S-sulfinate, in a time-dependent manner (Figure S1). These data suggest that in *E. histolytica* L-cysteine is likely involved in the scavenging of oxygen free radicals. The concomitant decrement in L-cystine also highlights the significance of NADPH-dependent oxidoreductase, which functions as cystine reductase to replenish L-cysteine [55]. We also observed a time-dependent increment in S-methylcysteine, which is synthesized from O-acetylserine and methanethiol [30]. Synthesis of S-methylcysteine upon oxidative stress suggests activation of serine acetyltransferases (SAT1 and SAT2) due to reversal of their inhibition by L-cysteine [56]. As mentioned above, the level of O-phosphoserine, a precursor of L-serine and L-cysteine [51], [52], was significantly increased in a time-dependent manner upon oxidative stress, whereas that of L-serine remained unchanged. Since O-phosphoserine phosphatase, an enzyme that converts O-phosphoserine to L-serine, has not been identified in *E. histolytica*, serine biosynthesis may not occur under the conditions used in the study. Thus, the fate of O-phosphoserine in *E. histolytica* still remains to be established. Apart from these limited changes, we did not observed any significant change in other amino acids.

### Oxidative stress inactivates key metabolic enzymes

Glycolytic enzymes are known to be highly susceptible to inhibition by reactive oxygen species [57], [58]. We assayed activities of several key enzymes of glycolysis and its associated pathways upon PQ-mediated oxidative stress. Among the tested enzymes, the activities of PFOR, phosphoglycerate mutase (PGAM), and NAD^+^ dependent alcohol dehydrogenase (ADH) were decreased to 33±21, 46±15, and 61±21%, respectively. However, the activities of GAPDH, TPI, PGK, ENO, and PPDK were decreased by only 16–22%, and the activities of remaining enzymes were not significantly changed (Fig. 4A). PFOR and ADH have previously been shown to be inactivated by O_2_-mediated oxidative stress in *E. histolytica* [22]. In addition to PFOR, we also found that PGAM was inactivated by PQ-mediated superoxide stress. However, PGAM was not found to be inhibited by O_2_-mediated oxidative stress [20]. Thus inhibition of PGAM may be specific to PQ generated superoxide radicals. As PGAM is a glycolytic flux-regulating enzyme in *E. histolytica* [59], its inactivation will have more effect on glycolytic activity, and hence cause the redirection of flux towards associated pathways. Thus, drastic inhibition of PGAM, PFOR and ADH, and partial inhibition of other glycolytic enzymes resulted in the overall reduction in glycolytic flux, and accumulation of glycolytic intermediates.

### Redirection of metabolic flux towards glycerol production upon oxidative stress

Identification of glycerol 3-phosphate dehydrogenase (G3PDH) and glycerol kinase (GK) in the genome of *E. histolytica* suggests the presence of glycerol metabolism in this parasite [12]. However, the activity of G3PDH in the soluble fraction of *E. histolytica* was not detected by conventional enzymatic methods [60], and it was proposed that dihydroxyacetone phosphate (DHAP) is mainly used for triglyceride synthesis, but not glycerol 3-phosphate (G 3-P). In contrast, as shown in Fig. 2, we found Gly 3-P as one of the most highly modulated (10–14 fold) metabolites upon oxidative stress. The drastic accumulation of Gly 3-P, together with the increase in other upstream glycolytic intermediates, upon oxidative stress clearly suggests the presence of functional G3PDH in this parasite. We also tested if accumulated Gly 3-P is also converted to glycerol leading to its accumulation. As shown in Fig. 4B, the level of intracellular glycerol was also dramatically increased upon PQ-mediated oxidative stress. As the changes in the levels of Gly 3-P and glycerol were similar in terms of fold changes, most of Gly 3-P produced upon oxidative stress is likely converted to glycerol. We also assayed production of ethanol and acetate upon PQ-mediated oxidative stress, and found that ethanol production was significantly decreased, while the acetate level remained unchanged (Fig. 4, C and D). These data indicate that *E. histolytica* is capable of glycerol biosynthesis from glucose, similar to other protozoa such as *T. vaginalis*, *Trypanosoma brucei*, and *Plasmodium falciparam* [61]–[63].

**Figure 5:**
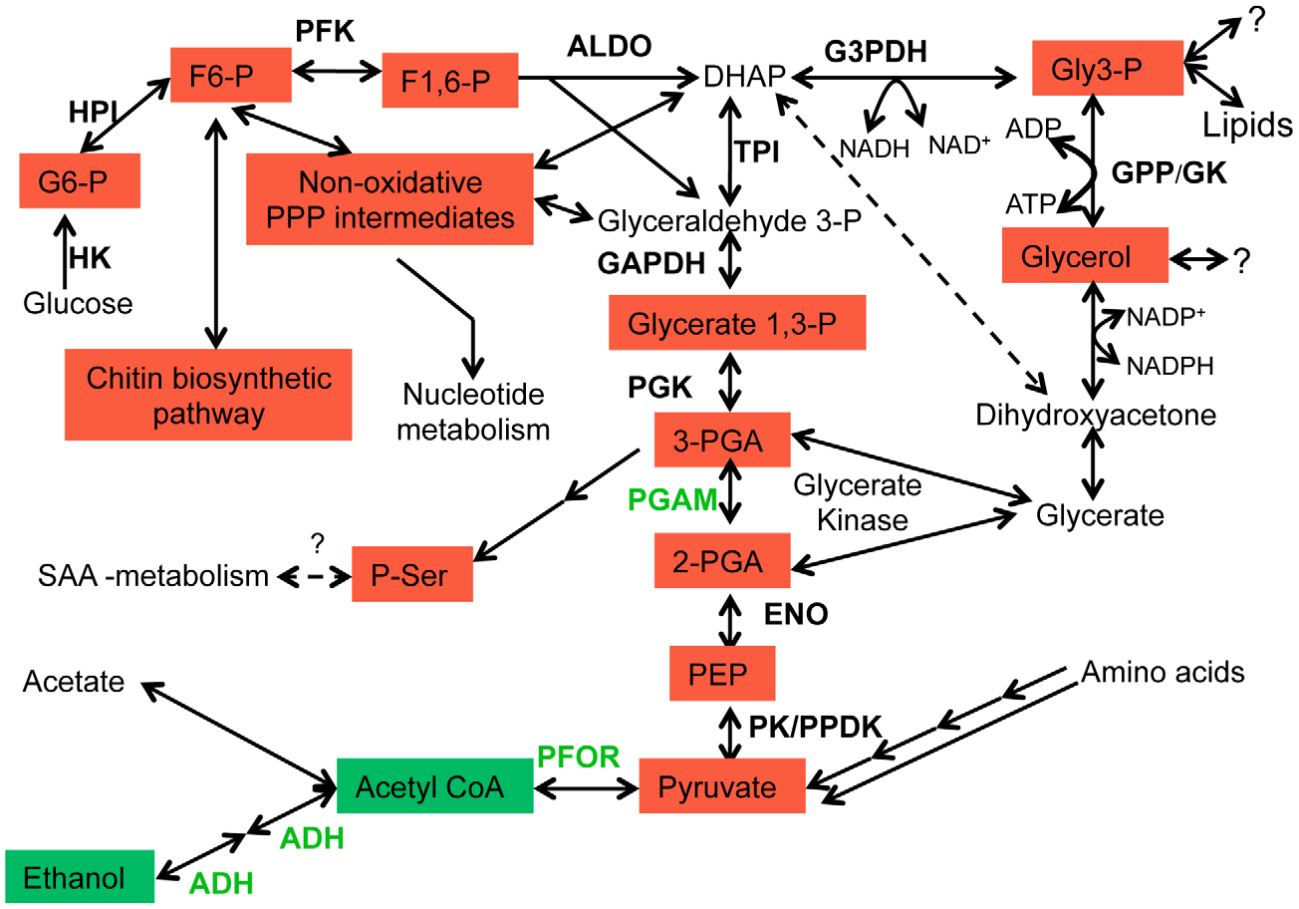
Representation of major changes in central carbon metabolism upon oxidative stress. Shades in red and green indicate increase or decrease of metabolites, respectively. Enzymes catalyzing these reactions are also shown in bold letters. Enzymes in black or green are those whose activities were unchanged or downregulated, respectively, upon oxidative stress. Abbreviations are: HK, Hexokinase; HPI, Hexose phosphate isomerase; PFK, Phosphofructokinase; ALDO, Aldolase; TPI, Triose-phosphate isomerase; GAPDH, glyceraldehyde 3-P dehydrogenase; PGK, Phosphoglycerate kinase; ENO, Enolase; PPDK, Pyruvate phosphate dikinase; PK, Pyruvate kinase; MDH, Malate dehydrogenase; ME, malic enzyme; ADH, Alcohol dehydrogenase; GK, glycerol kinase; GPP, glycerol 3-phosphate phosphatase.

While importance of glycerol metabolism in oxidative stress defense has previously been shown [64], its specific induction upon oxidative stress was not reported. In yeast, it was shown that Gly 3-P was converted to glycerol by the action of Gly 3-P phosphatase (GPP), and deletion of this gene resulted in the increased sensitivity of yeast to peroxide and superoxide stress [64]. Although glycerol functions as an efficient free radical scavenger, other mechanisms by which glycerol protects against oxidative stress are equally possible. One of these alternative mechanisms is the NADP-dependent conversion of glycerol to dihydroxyacetone (by glycerol dehydrogenase) or glyceraldehyde (by glyceraldehyde reductase), leading to the formation of NADPH (Fig. 5). DHAP generated in this reaction may then be converted to dihydroxyacetone phosphate. Glyceraldehyde can be converted to glycerate, and subsequently enter glycolysis as 2-phosphoglycerate bypassing PGAM, which is one of the flux-controlling enzymes targeted by free radicals (Fig. 4A, and Fig. 5). We also showed that increment in Gly 3-P also led to equivalent increment in glycerol. Two enzymes, GPP and GK, may catalyze the conversion of Gly 3-P to glycerol (Fig. 5). As *E. histolytica* lacks GPP, GK is likely responsible for the conversion, which generates ATP (Fig. 5). Thus, an alternative role of redirection of metabolic flux towards glycerol may be to generate energy from accumulated glycolytic intermediates. Conversion of one molecule of DHAP to ethanol or acetate generates 2 or 3 ATP molecules, respectively. In contrast, synthesis of glycerol from DHAP by the sequential action of G3PDH and GK generates only one ATP molecule. Thus, redirection of glycolytic flux towards glycerol reduces efficiency of energy generation, but may still be beneficial for the trophozoites to extract the energy contained in the accumulated glycolytic intermediates upon oxidative stress. Further studies are needed to precisely determine the role of glycerol metabolism in generation of ATP and reducing powers under oxidative stress in *E. histolytica*.

## Conclusions

In the present study, we demonstrated that exposure to oxidative stress led to drastic metabolic changes in *E. histolytica*. While the expression of genes involved in the metabolism was only slightly affected by oxidative stress, several key enzymes involved in glycolysis were inactivated to re-direct the metabolic flux towards the associated pathways such as the non-oxidative branch of PPP, chitin, and glycerol biosynthetic pathways (Fig. 5). We also demonstrated the functionality of G3PDH, and showed, like other protozoan parasites, that GK in *E. histolytica* is capable of catalyzing the reverse reaction, leading to formation of glycerol and ATP (Fig. 5). Detailed biochemical and functional analysis of G3PDH and GK is necessary to understand the role of glycerol metabolism in the energy metabolism and oxidative stress tolerance. It is also important to know the fate and physiological significance of increased production of glycerol 3-phosphate and glycerol upon oxidative stress.

## Supporting Information

Figure S1 **Oxidative stress modulates amino acid metabolism.** The average fold change ± SD (error bars) of metabolites in untreated (control), H_2_O_2_, or PQ treated trophozoites with respect to time 0 h is shown. Abbreviations are: SAM, S-adenosylmethionine; SAH, S-adenosylhomocysteine; dcSAMd, decarboxylated S-adenosylmethionine; 5-MTA, 5′-methylthioadenosine; PEP, Phosphoenolpyruvate; MetSO, Methionine sulfoxide; OAS, O-acetylserine; SMC, S-methylcysteine; P-Ser, O-phosphoserine; 3-PGA, 3-phosphoglycerate.
(TIF)
