# Supplementary figures and images for: Dramatic Increase in Glycerol Biosynthesis upon Oxidative Stress in the Anaerobic Protozoan Parasite Entamoeba histolytica
Source: PLoS Negl Trop Dis. 2012 Sep 27;6(9):e1831. doi: 10.1371/journal.pntd.0001831 (PMC3459822; doi:10.1371/journal.pntd.0001831)

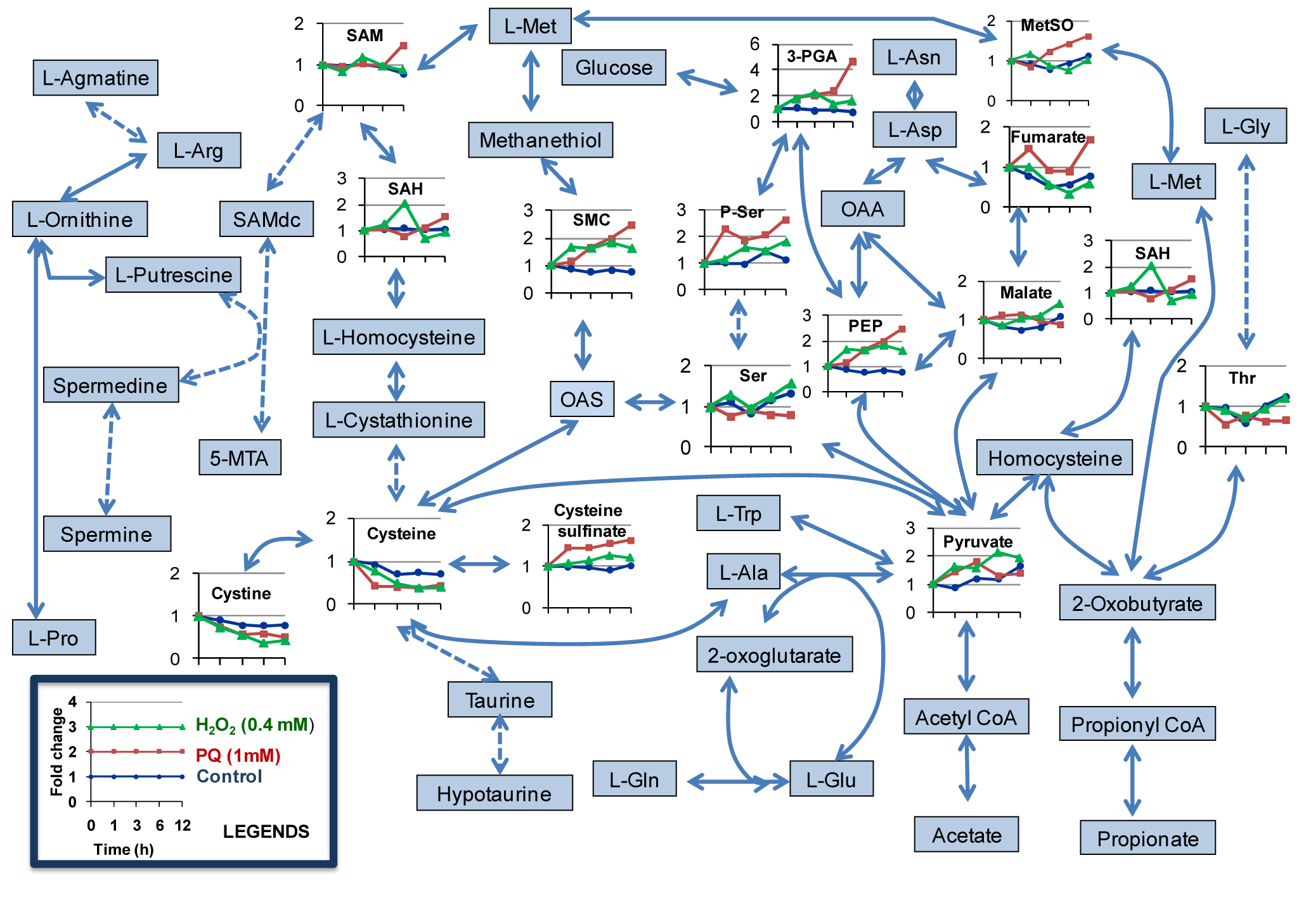

Supplement: Figure S1 — Oxidative stress modulates amino acid metabolism. The average fold change ± SD (error bars) of metabolites in untreated (control), H2O2, or PQ treated trophozoites with respect to time 0 h is shown. Abbreviations are: SAM, S-adenosylmethionine; SAH, S-adenosylhomocysteine; dcSAMd, decarboxylated S-adenosylmethionine; 5-MTA, 5′-methylthioadenosine; PEP, Phosphoenolpyruvate; MetSO, Methionine sulfoxide; OAS, O-acetylserine; SMC, S-methylcysteine; P-Ser, O-phosphoserine; 3-PGA, 3-phosphoglycerate. (TIF) [file pntd.0001831.s001.tif]
